# Long noncoding RNA BACE1-antisense transcript plays a critical role in Parkinson’s disease via microRNA-214-3p/Cell death-inducing p53-target protein 1 axis

**DOI:** 10.1080/21655979.2022.2066750

**Published:** 2022-04-28

**Authors:** Lina Li, Hongjuan Wang, Huicang Li, Xin Lu, Yanxiang Gao, Xiaofeng Guo

**Affiliations:** a First Department of Neurology, Baoji Hospital of Traditional Chinese Medicine, Baoji, Shaanxi, China; b Second Department of Neurology, Baoji Hospital of Traditional Chinese Medicine, Baoji, Shaanxi, China; c First Department of Neurology, Yangquan Third People’s Hospital, Yangquan Shaanxi, Yangquan, Shaanxi, China; d Department of Neurology, Baoji People’s Hospital, Baoji, Shaanxi, China

**Keywords:** Long noncoding RNA (lncRNA) BACE1-antisense transcript (BACE1-AS), parkinson’s disease, microRNA-214-3p, oxidative stress, inflammatory response

## Abstract

This study aimed to analyze the function and latent mechanism of long noncoding RNA BACE1-antisense transcript (lncRNA BACE1-AS) in MPP^+^-induced SH-SY5Y cells. SH-SY5Y cells were cultivated in 1 mM MPP^+^ for 24 h to establish Parkinson’s disease (PD) model *in vitro*. TargetScan and luciferase reporter assay were conducted to predict and verify the interaction between microRNA (miR)-214-3p and CDIP1 (Cell death-inducing p53-target protein 1). Cell viability, lactate dehydrogenase (LDH) release, and cell apoptosis were evaluated by 3-(4,5-dimethylthiazol-2-yl)-2,5-diphenyl-2 H-tetrazolium bromide (MTT), LDH, and flow cytometer. The secretion of inflammatory factors and representative biomarkers of oxidative stress, including reactive oxygen species (ROS) and superoxide dismutase (SOD) were assessed using enzyme-linked immunosorbent assay (ELISA) and specific assay kits. Results suggested that lncRNA BACE1-AS was over-expressed and miR-214-3p was under-expressed in MPP^+^-stimulated SH-SY5Y cells. Further analyses revealed that MPP^+^ inhibited cell viability; enhanced cell apoptosis, Cleaved Caspase-3 expression and Cleaved Caspase-3/GAPDH ratio; induced oxidative stress and inflammation in SH-SY5Y cells were inhibited by lncRNA BACE1-AS-siRNA transfection; and all these inhibitions were reversed by miR-214-3p inhibitor. In addition, we found that CDIP1 was directly targeted by miR-214-3p and up-regulated in MPP^+^-stimulated SH-SY5Y cells. Further functional assays suggested that CDIP1-plasmid reversed the effects of miR-214-3p mimic on MPP^+^-stimulated SH-SY5Y cells. In conclusion, lncRNA BACE1-AS regulates SH-SY5Y cell proliferation, apoptosis, inflammatory response, and oxidative stress through direct regulation of miR-214-3p/CDIP1 signaling axis, and could be a potential candidate associated with the diagnosis and treatment of PD.

## Graphical abstract

**Figure:**
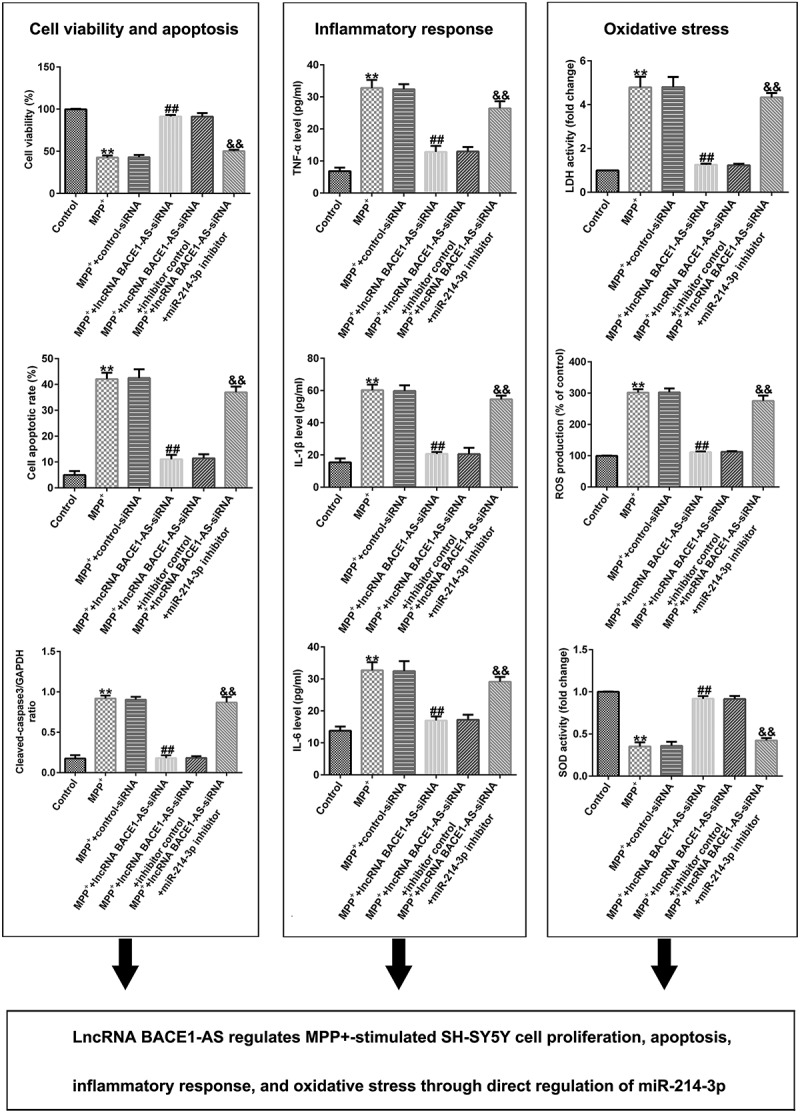

## Research highlights

- LncRNA BACE1-AS was over-expressed and miR-214-3p was under-expressed in MPP^+^-induced SH-SY5Y cells;
- Down-regulation of miR-214-3p inverted the influence of lncRNA BACE1-AS-siRNA on MPP^+^-induced SH-SY5Y cells;
- CDIP1-plasmid inverted the functions of miR-214-3p mimic in MPP^+^-induced SH-SY5Y cells.

## Introduction

Parkinson’s disease (PD) is a frequently occurring neurodegenerative disease in the middle-aged and older adults. The prevalence of PD in people over 65 years old in China is about 1.7% [1,2]. Most patients with Parkinson’s disease are sporadic cases, and less than 10% of patients have a family history [3]. Many elements are involved in the degeneration and death of PD dopaminergic neurons, including genetic factors, environmental factors [4], aging, and oxidative stress [5]. For example, Liu et al studied the function of moxibustion in alleviating oxidative stress damage in PD rats through Nrf2/ARE pathway [6]. However, the exact cause of this pathological change is still unclear. Therefore, illustrating the mechanisms responsible for PD treatment might require a novel strategy.

LncRNA is a non-coding RNA, which is a group of RNA with a transcription length of more than 200 nucleotides and low translation ability [7]. Previous reports have suggested that lncRNAs were related to the regulation of various biological processes, including transcription, protein degradation or adsorbing miRNAs [8,9]. Several studies have indicated that lncRNA is closely associated with the occurrence and development of PD. Chen et al. suggested that MPP^+^ significantly upregulated the levels of lncRNA MALAT1 in PD model *in vitro* [10]. By analyzing the lncRNA expression in patients with PD, it was found that lncRNA HOX antisense intergenic RNA (HOTAIR) accelerated MPP^+^-stimulated neuronal damage in PD via adjusting miR-874-5p/ATG10 axis, indicating that it may be closely related to the pathological changes of PD [11]. Previous investigations have suggested that lncRNA BACE1-AS is significantly up-regulated in PD [12]; however, its specific role and molecular mechanisms are still unclear.

The miRNA, a non-coding RNAs with a length of 20 nucleotides, acts as a transcriptional regulator in various physiological progresses [13]. Currently, multiple miRNAs have been evidenced to be regulated in human brain tissues, including PD. Tao et al. suggested that miR-384-5p adjusted PD progression via regulating SIRT1 in mice and SHSY5Y cells [14]. MiR-214-3p has been shown as a vital carcinogenic gene for many common cancers, such as bladder [15] and breast cancer [16]. Moreover, miR-214-3p was significantly down-regulated in patients with PD [17], but its role and mechanism remain unclear. Recent research shows that lncRNA BACE1-AS sponges to miR-214-3p [18].

Therefore, in this study, we hypothesized that (i) lncRNA BACE1-AS may be a vital regulator in the development of PD; and (ii) the underlying mechanisms of lncRNA BACE1-AS’s protective effects might be relevant to miR-214-3p/CDIP1 axis. This study aimed to analyze the roles of lncRNA BACE1-AS in patients with PD and elucidate its latent mechanisms in regulating miR-214-3p/CDIP1 axis. The findings of this study revealed that lncRNA BACe1-AS regulates oxidative stress in inflammatory response and neuronal apoptosis via regulating mir-214-3p/CDIP1 signaling axis in the progression of PD, indicating that lncRNA BACE1-AS might be a promising biomarker for PD diagnosis and treatment.

## Materials and methods

### Cell culture and establishment of PD model

The SH-SY5Y cells were acquired from American Type Culture Collection and maintained in RPMI-1640 medium (cat. no. 11,875,093; Gibco, USA) containing 15% FBS, 1% streptomycin and penicillin at 37°C in a humidified atmosphere containing 5% CO_2_. The SH-SY5Y cells were cultivated in 1 mM MPP^+^ (cat. no. 36,913-39-0; Sigma, USA) for 24 hours to build PD cell model *in vitro* [19].

### qRT-PCR analysis

After treatment, the level of lncRNA BACE1-AS and miR-214-3p were assessed using qRT-PCR. Total RNA was obtained from SH-SY5Y cells using RNA-isolation kit (cat. no. 9108; Takara, Beijing, China) in accordance with protocol. Then the total RNA was reversed to cDNA following the instructions of PrimeScript RT Reagent Kit (cat. no. RR037B; Takara) and qRT-PCR analysis was conducted using the DyNAmo HS SYBR Green qPCR Kit (cat. no. F410L, Thermo Scientific) with ABI 7500 Real-Time PCR System (Agilent Technologies, USA). The relative quantification of target genes was analyzed using 2^−ΔΔCt^ method [20]. Primer sequences were listed as following:

U6 forward, 5′-GCTTCGGCAGCACATATACTAAAAT-3′;

reverse, 5′-CGCTTCACGAATTTGCGTGTCAT-3′;

GAPDH forward, 5′-CTTTGGTATCGTGGAAGGACTC-3′;

reverse, 5′-GTAGAGGCAGGGATGATGTTCT-3′;

lncRNA BACE1-AS forward, 5′-GGCACCTCCTAAGTGTACCTGC-3′;

reverse, 5′-CTCTCTGCTGGGCACGATTC-3′;

miR-214-3p forward, 5′-GCATCCTGCCTC CACATGCAT-3′;

reverse, 5′-GCGCTGAGGAATAATAGA GTATGTAT-3′;

CDIP1 forward, 5′-GACTTCAGCCTTTTGTTCATGG-3′;

reverse, 5′-TCTTTGCTGTTGATACTCCTGG-3′.

### Cell transfection

The control-siRNA (5’-GCGCGATAGCGCGAATATA-3’; Sangon Biotech), lncRNA BACE1-AS-siRNA (5’-AGAAGGGTCTAAGTGCAGACATCTG-3’; Sangon Biotech), inhibitor control (5’-CAGTACTTTTGTGTAGTACAA-3’; Gene Pharma, Shanghai, China), miR-214-3p inhibitor (5’-ACTGCCTGTCTGTGCCTGCTGT-3’; Gene Pharma, Shanghai, China), mimic control (5’-UUCUCCGAACGUGUCACGUTT-3’; Gene Pharma, Shanghai, China), miR-214-3p mimic (5’-ACAGCAGGCACAGACAGGCAGU-3’; Gene Pharma, Shanghai, China), and control-plasmid (cat. no. sc-437,275; Santa Cruz Biotechnology) or CDIP1-plasmid (cat. no. sc-409,425-ACT; Santa Cruz Biotechnology) were transfected into SH-SY5Y by Lipofectamine™3000 (Life Technologies) in accordance with protocol.

### MTT assay

SH-SY5Y cells were cultured into 96-well plates. Then, cells were treated with 10μl MTT (5 mg/ml) solution and cultivated for 4 h. Then dimethyl sulfoxide was added to plates, which were kept in the dark for 10 min thereafter. Finally, the OD_570 nm_ value was measured using a microplate reader (BioTek, USA) after vibration mixing following the instructions [21].

### Flow cytometry analysis

After treatment, SH-SY5Y cells were analyzed using an annexin V-FITC/PI apoptosis detection kit (cat. no. C1062S; Beyotime, Shanghai, China) for 30 min at 37°C in the dark per the instructions. Then apoptotic cells were identified by flow cytometry and analyzed using Kaluza analysis software (version 2.1.1.20653; Beckman Coulter, Inc.) [22].

### Western blot assay

Protein levels were measured using Western blot assay [23]. Total proteins were acquired using RIPA lysis buffer (cat. no. P0013B; Beyotime, Shanghai, China), and protein concentration was measured using a BCA Protein Assay kit (cat. no. BCA1; Sigma, MO, USA). Then protein samples were separated using 10% SDS-PAGE and blotted onto PVDF membrane. After sealing with 5% non-fat milk in PBST for 1h, the membranes were incubated with primary antibodies against GAPDH, CDIP1, and Cleaved Caspase-3 (1:1000 dilution) overnight at 4° C. After washing, the membranes were treated with secondary antibodies for 1 h. Finally, the protein bands were visualized using ECL detection system reagents (Pierce, USA) and quantified using ImageJ software version 1.8.0 (NIH, Bethesda, MD, USA).

### Lactate dehydrogenase (LDH) assay

LDH released from SH-SY5Y cells were detected using a LDH reagent kit (cat. no. 11,644,793,001; Sigma) [19]. Briefly, the supernatant of SH-SY5Y cells were collected from each well after treatment. Then, the culture supernatant and cell lysates were cultivated with LDH reaction mixture following the manufacturer’s protocol for 15 min. The OD_490nm_ was determined and LDH release was calculated with a microplate reader (BioTek, USA).

### ELISA assay

After treatment, SH-SY5Y cells were centrifuged for 10 min; then tumor necrosis factor-α (TNF-α; cat. no. 555,212) and IL-1β (cat. no. 557,953) levels in cells culture supernatant were detected using ELISA kits (BD Sciences) following the instructions. The OD_450_ was measured using Multiscan Spectrum (MD, USA) referring to the product instructions [19].

### Measurement of reactive oxygen species (ROS) and superoxide dismutase (SOD) levels

After treatment, SH-SY5Y cells were induced by 1 mM MPP^+^ for 24 h. Subsequently, the levels of SOD and ROS in the supernatant were checked using the Total Superoxide Dismutase Assay Kit (cat. no. S0101S; Beyotime, China) and ROS Assay Kit (cat. no. S0033S; Beyotime, China), respectively [24].

### Dual-luciferase reporter assay

The relationship between miR-214-3p and CDIP1 was predicted using TargetScan (https://www.targetscan.org/vert_80/). The CDIP1 3’-UTR, which includes the miR-214-3p binding site or a mutated target site, was amplified and fixed onto pMIR vectors (Ambion, USA) to obtain wild-type CDIP1 plasmid (CDIP1-WT) or CDIP1 mutated plasmid (CDIP1-MUT). The CDIP1-WT or CDIP1-MUT and miR-214-3p mimics or mimic control were co-transfected into 293T cell by Lipofectamine 2000 and cultivated for 48 h following the instructions. Dual-Luciferase Reporter Assay System (cat. no. E1910; Promega) was applied to check the luciferase activity [25].

### Statistical analysis

All statistical analyses were performed using SPSS 19.0 (SPSS Inc., Chicago, IL). All results are expressed by mean ± SD from three independent experiments. The mean diversity between groups was estimated by one-way ANOVA with Tukey’s post hoc test or Student’s t-test. *P < 0.05, and **P < 0.01 were indicated as significant difference.

## Results

### LncRNA BACE1-AS was over-expressed and miR-214-3p was down-regulated in MPP^+^-induced SH-SY5Y cells

**Figure 1.**
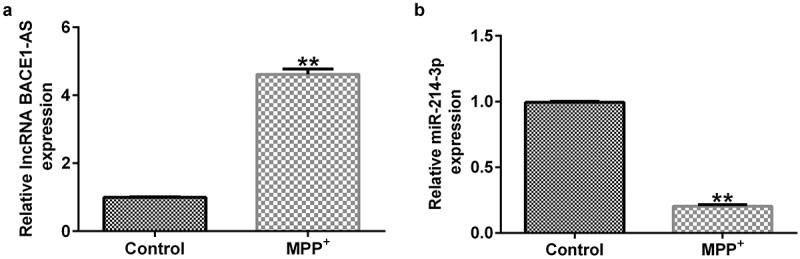
*Expression of lncRNA BACE1-AS and miR-214-3p in SH-SY5Y cells*. qRT-PCR analysis of lncRNA BACE1-AS (a) and miR-214-3p (b) expression in SH-SY5Y cells. * P < 0.05, **P < 0.01 vs. control.

Previous reports have suggested that lncRNA BACE1-AS participated in Alzheimer’s disease by regulating miR-214-3p. Moreover, miR-214-3p has been evidenced to be dramatically down-regulated in patients with PD. We evaluated the lncRNA BACE1-AS and miR-214-3p levels in SH-SY5Y cells using qRT-PCR after establishing the PD models. The expression of lncRNA BACE1-AS was higher in MPP^+^-stimulated SH-SY5Y cells than that in control group (Figure 1a; p < 0.01). Moreover, miR-214-3p expression was down-regulated after MPP^+^ treatment (p < 0.01). Our results suggested that lncRNA BACE1-AS and miR-214-3p was associated with the occurrence and progression of PD.

### miR-214-3p inhibitor reverses the influence of lncRNA BACE1-AS-siRNA on miR-214-3p levels in SH-SY5Y cells

**Figure 2.**
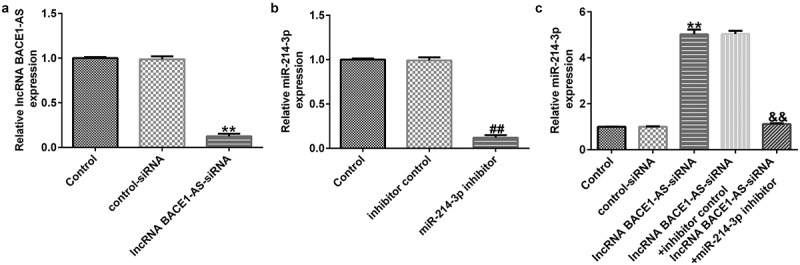
*Effect of lncRNA BACE1-AS-siRNA or miR-214-3p inhibitor on miR-214-3p levels in SH-SY5Y cells*. Control-siRNA, lncRNA BACE1-AS-siRNA, inhibitor control, miR-214-3p inhibitor were applied to stimulate SH-SY5Y cells for 48 h. (a) qRT-PCR analysis of lncRNA BACE1-AS in lncRNA BACE1-AS-siRNA or control-siRNA transfected SH-SY5Y cells. (b) The inhibitory efficiency of miR-214-3p inhibitor on miR-214-3p level was checked using qRT-PCR. (c) Determination of miR-214-3p level in BACE1-AS-siRNA+ inhibitor control or BACE1-AS-siRNA+ miR-214-3p inhibitor transfected SH-SY5Y cells using qRT-PCR. * P < 0.05, **P < 0.01 vs. control.

To determine the effects of lncRNA BACE1-AS on miR-214-3p expression in SH-SY5Y cells, SH-SY5Y cells were treated with control-siRNA, lncRNA BACE1-AS-siRNA, inhibitor control or miR-214-3p inhibitor, and levels were evaluated using qRT-PCR assay. Our data suggested that lncRNA BACE1-AS-siRNA remarkably reduced lncRNA BACE1-AS expression in SH-SY5Y cells, compared with control-siRNA (Figure 2a; p < 0.01). Meanwhile, miR-214-3p was under-expressed in miR-214-3p inhibitor transfected SH-SY5Y cells, in comparison with inhibitor control group (Figure 2b; p < 0.01). Besides, in comparison with control-siRNA group, lncRNA BACE1-AS-siRNA up-regulated miR-214-3p mRNA levels in SH-SY5Y cells. Nevertheless, this effect of lncRNA BACE1-AS-siRNA on miR-214-3p was reversed by miR-214-3p inhibitor in SH-SY5Y cells (Figure 2c; p < 0.01). In conclusion, our observations indicated that miR-214-3p may interfere with lncRNA BACE1-AS functions in patients with PD.

### Down-regulation of miR-214-3p inverted the influence of lncRNA BACE1-AS-siRNA on SH-SY5Y growth and apoptosis

**Figure 3.**
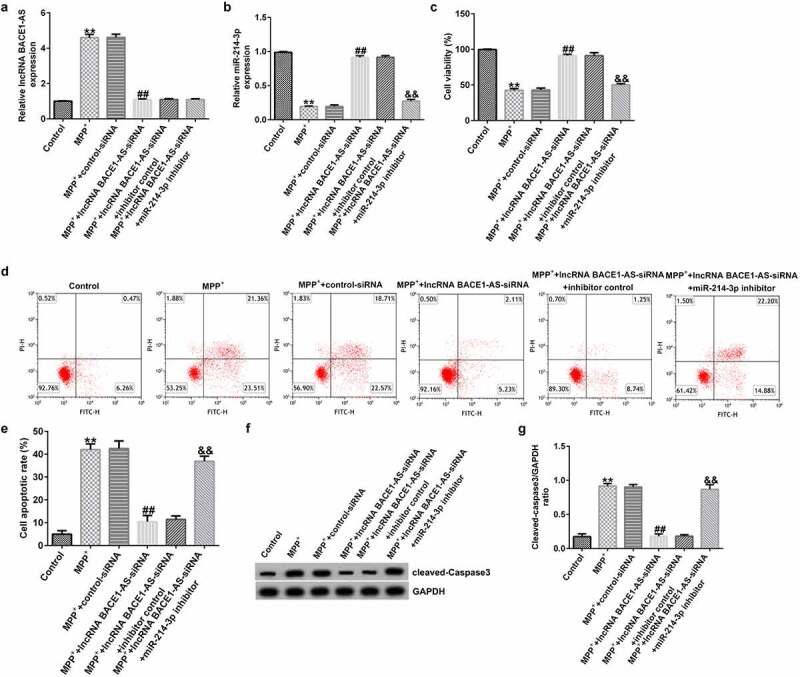
*Effects of lncRNA BACE1-AS-siRNA on cells growth and apoptosis in MPP^+^ stimulated cells*. SH-SY5Y cells were induced by control-siRNA, lncRNA BACE1-AS-siRNA, and BACE1-AS-siRNA+ inhibitor control or BACE1-AS-siRNA+miR-214-3p inhibitor for 48 h and exposed to 1 mM MPP^+^ for 24 h. qRT-PCR analysis of lncRNA BACE1-AS (a) and miR-214-3p (b) levels in SH-SY5Y cells in different groups. (c) MTT assay was employed to check cell viability. (d) Apoptotic SH-SY5Y cells were evaluated using flow cytometry analysis. (e) Analysis of apoptotic cells. (f) Western blot analysis of Cleaved Caspase-3 expression. (g) The ratio of Cleaved Caspase-3/GAPDH value. * P < 0.05, **P < 0.01 vs. control.

To further understand the roles of miR-214-3p in PD model *in vitro*, control-siRNA, lncRNA BACE1-AS-siRNA, and inhibitor control or miR-214-3p inhibitor were added into SH-SY5Y cells for 48 h and exposed to 1mM MPP^+^ for 24 h. Our findings demonstrated that lncRNA BACE1-AS levels was higher in MPP^+^-induced SH-SY5Y cells (Figure 3a; p < 0.01), in comparison with control group. Moreover, miR-214-3p level was lower in MPP^+^-stimulated SH-SY5Y cells, in comparison with control group (Figure 3b; p < 0.01). We also analyzed SH-SY5Y cells viability and apoptosis in different groups. MPP^+^ stimulation led to inhibition of cell viability and induction of apoptotic cells (Figure 3(c-e)). Furthermore, Cleaved Caspase-3 expression and Cleaved Caspase-3/GAPDH ratio (figure 3(f-g)) in MPP^+^ group were higher than those in control group. However, we observed the opposite findings in MPP^+^ lncRNA BACE1-AS-siRNA group, compared to MPP^+^ control-siRNA, and these observations were inverted after miR-214-3p inhibitor treatment. Therefore, these results suggested that miR-214-3p inhibitor inverted the effects of lncRNA BACE1-AS-siRNA on SH-SY5Y viability and apoptosis.

### miR-214-3p inhibitor inverted the influence of lncRNA BACE1-AS-siRNA on MPP^+^ stimulated oxidative stress and inflammation in SH-SY5Y cells

**Figure 4.**
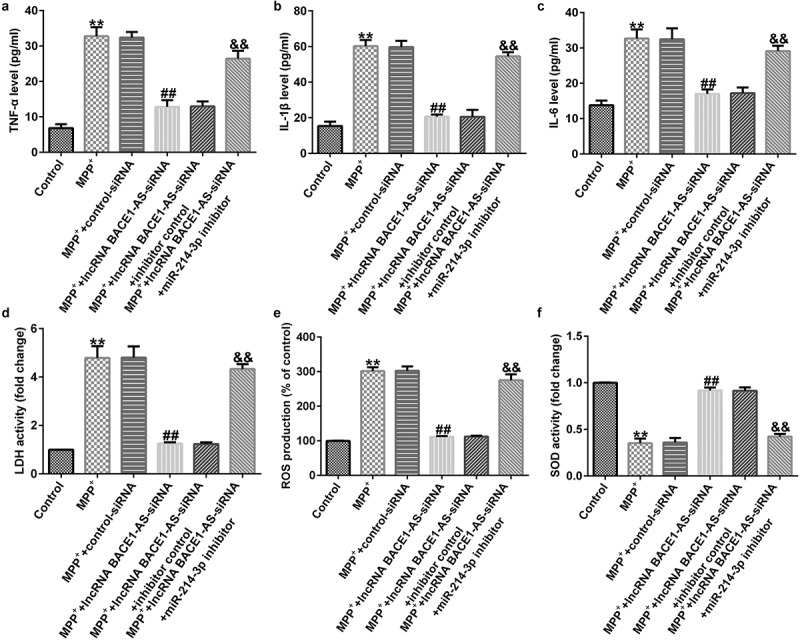
*Effects of lncRNA BACE1-AS-siRNA on inflammatory response and oxidative stress in MPP^+^-stimulated cells*. Levels of tumor necrosis factor-α (TNF-α) (a), IL-1β (b), and IL-6 (c) in SH-SY5Y cell supernatant were determined using ELISA. (d) Detection of lactate dehydrogenase (LDH) release. (e and f) Reactive oxygen species (ROS) and superoxide dismutase (SOD) activity in different groups were assessed. * P < 0.05, **P < 0.01 vs. control.

Furthermore, we explored the roles of lncRNA BACE1-AS-siRNA in LDH leakage, oxidative stress, and inflammation in MPP^+^ stimulated SH-SY5Y cells. MPP^+^ significantly promoted the secretion of TNF-α, IL-1β and IL-6 (Figure 4(a-c); all p < 0.01). Moreover, MPP^+^ significantly promoted LDH release (Figure 4d; p < 0.01), enhanced ROS (Figure 4e; p < 0.01) and inhibited the activity of SOD (figure 4f; p < 0.01). Nevertheless, compared to MPP^+^+control-siRNA group, we observed the opposite results in MPP^+^+lncRNA BACE1-AS-siRNA group, and all the effects of lncRNA BACE1-AS-siRNA on MPP^+^ stimulated SH-SY5Y cells were reversed by miR-214-3p inhibitor. In summary, our data indicated that lncRNA BACE1-AS-siRNA prevents MPP^+^ induced oxidative stress and inflammation by regulating miR-214-3p.

### Interaction of CDIP1 with miR-214-3p

**Figure 5.**
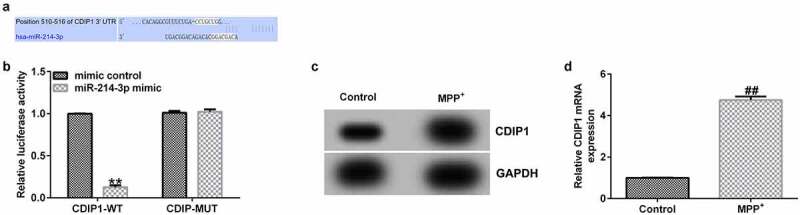
*CDIP1 directly targeted miR-214-3p*. (a) Relationship between lncRNA BACE1-AS and miR-214-3p was predicted using TargetScan. (b) Dual-luciferase reporter gene system analysis evidenced that CDIP1 directly targeted miR-214-3p. (c-d) qRT-PCR and Western blot analysis of CDIP1 in SH-SY5Y cells. * P < 0.05, **P < 0.01 vs. control.

Having investigated the relevance between lncRNA BACE1-AS and miR-214-3p, we analyzed the latent mechanisms of miR-214-3p in SH-SY5Y cells. The bioinformatics prediction was made by TargetScan. CDIP1 was a potential target of miR-214-3p (Figure 5a). Furthermore, dual-luciferase reporter system affirmed the relevance between CDIP1 and miR-214-3p. We found that up-regulation of miR-214-3p suppressed luciferase activity of CDIP1-WT reporter but caused no changes in CDIP1-MUT group (Figure 5b; p < 0.01). In addition, results from Western blot and qRT-PCR analysis demonstrated that CDIP1 was over-expressed in MPP^+^ treated SH-SY5Y cells (Figure 5(c,d); all p < 0.01), indicating that CDIP1 directly interacted with miR-214-3p.

### miR-214-3p regulated CDIP1 levels in SH-SY5Y cells

**Figure 6.**
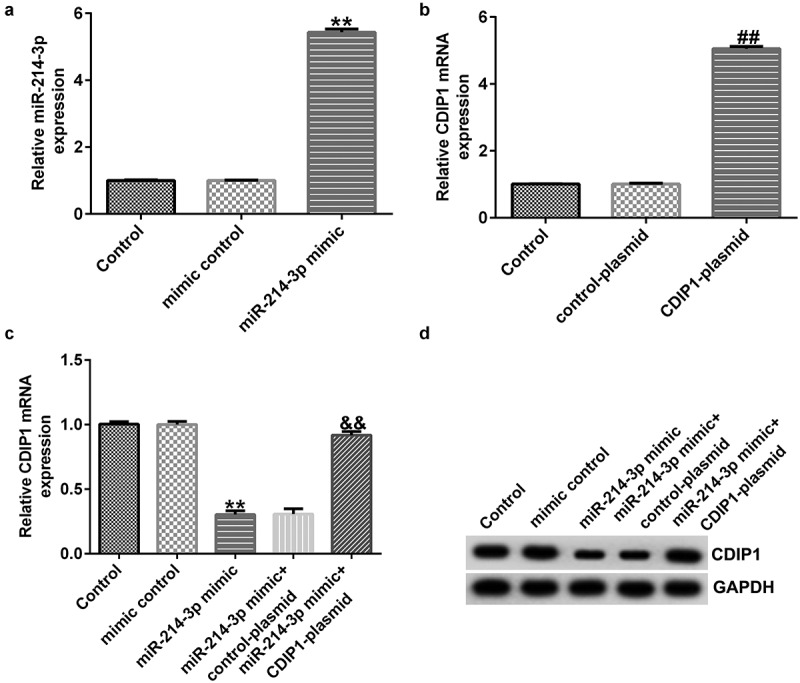
*Influences of miR-214-3p mimic or CDIP1-plasmid on CDIP1 expression in SH-SY5Y cells*. (a) qRT-PCR analysis of miR-214-3p level in mimic control and miR-214-3p mimic group. (b) Effect of CDIP1-plasmid or control-plasmid on CDIP1 mRNA levels was measured by qRT-PCR. (c-d) Determination of CDIP1 expression in miR-214-3p mimic or miR-214-3p mimic+ CDIP1-plasmid transfected SH-SY5Y cells. * P < 0.05, **P < 0.01 vs. control.

To determine the effects of miR-214-3p on CDIP1 expression in SH-SY5Y cells, mimic control, miR-214-3p mimic, and control-plasmid or CDIP1-plasmid were transfected into SH-SY5Y cells for 48 h. In comparison with mimic control group, miR-214-3p mimic dramatically raised the level of miR-214-3p (Figure 6a; p < 0.01). We observed that CDIP1 was up-regulated in CDIP1-plasmid transfected SH-SY5Y cells, in comparison with control-plasmid (Figure 6b; p < 0.01). In addition, miR-214-3p mimic dramatically decreased CDIP1 mRNA levels and protein expression in SH-SY5Y cells compared to mimic control group. However, we observed the opposite results in CDIP1-plasmid co-transfected group, as evidenced by enhanced CDIP1 mRNA levels and protein expression (Figure 6(c,d); p < 0.01). Based on these data, we verified that miR-214-3p negatively regulated CDIP1 expression in SH-SY5Y cells.

### CDIP1-plasmid inverted the functions of miR-214-3p mimic in SH-SY5Y viability and apoptosis

**Figure 7.**
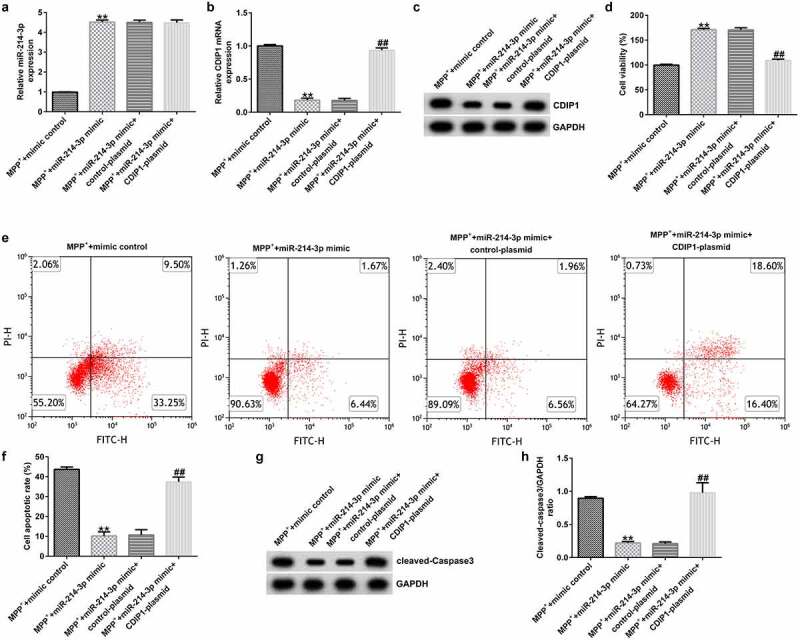
*miR-214-3p regulated SH-SY5Y cells viability and apoptosis via CDIP1*. miR-214-3p (a) and CDIP1 (b-c) levels in SH-SY5Y cells were assessed by qRT-PCR assay and Western blot. (d) MTT assay was applied to measure cell viability. (e) Analysis of apoptotic cells. (f) Quantification of apoptotic cells. (g) Determination of Cleaved Caspase-3 expression. (h) The ratio of Cleaved Caspase-3/GAPDH value. * P < 0.05, **P < 0.01 vs. control.

To explore the role of miR-214-3p and CDIP1 in MPP^+^ treated SH-SY5Y cells, miR-214-3p and CDIP1 expression were regulated in MPP^+^ treated SH-SY5Y cells. We observed that miR-214-3p was over-expressed (Figure 7a; p < 0.01) and CDIP1 was down-regulated in miR-214-3p mimic transfected and MPP^+^ treated SH-SY5Y cells (Figure 7(b,c); p < 0.01), compared to MPP^+^ mimic control group. We also explored SH-SY5Y cells growth and apoptosis using MTT and Flow cytometry (FCM) analysis. miR-214-3p mimic significantly promoted cell viability (Figure 7d; p < 0.01) and reduced cell apoptosis (Figure 7(e-f); p < 0.01) in MPP^+^ treated SH-SY5Y cells. Besides, Cleaved Caspase-3 expression (Figure 7g) and Cleaved Caspase-3/GAPDH ratio (Figure 7h; p < 0.01) in MPP^+^ miR-214-3p mimic group were higher than that in MPP^+^ mimic control group. Nevertheless, these changes were reversed by CDIP1-plasmid, suggesting that lncRNA BACE1-AS regulates neuronal growth and apoptosis by directly regulating miR-214-3p/CDIP1 signaling axis.

### CDIP1-plasmid inverted the functions of miR-214-3p mimic in oxidative stress and inflammation in SH-SY5Y cells

**Figure 8.**
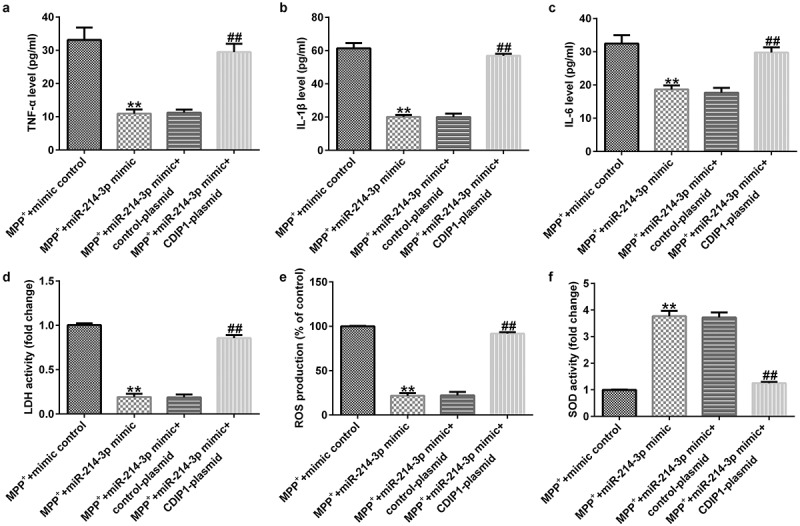
*miR-214-3p regulated SH-SY5Y cells inflammatory response and oxidative stress via CDIP1*. Levels of TNF-α (a), IL-1β (b), and IL-6 (c) in SH-SY5Y cell supernatant was determined by ELISA. (d) Detection of LDH release. (e and f) ROS and SOD activity in different groups were assessed. * P < 0.05, **P < 0.01 vs. control.

To further illustrate the potential mechanism of CDIP1, we evaluated the functions of CDIP1 in LDH leakage, oxidative stress, and inflammation in SH-SY5Y cells. Our data indicated that miR-214-3p mimic suppressed the release TNF-α, IL-1β, and IL-6 (Figure 8(a-c); all p < 0.01) in MPP^+^ stimulated SH-SY5Y cells. Furthermore, miR-214-3p mimic remarkably inhibited LDH release (Figure 8d; p < 0.01), reduced ROS levels (Figure 8e; p < 0.01) and raised SOD activity (figure 8f; p < 0.01) in MPP^+^ stimulated SH-SY5Y cells. However, we found contrary data in MPP^+^ miR-214-3p mimic^+^ CDIP1-plasmid group. Our findings indicated that lncRNA BACE1-AS regulates oxidative stress and inflammatory response by directly regulating miR-214-3p/CDIP1 signaling axis in SH-SY5Y cells.

## Discussion

PD is a common multifactorial neurodegenerative disease. Although there have been many reports and preclinical identification, latent pathogenesis and therapeutic strategies of PD have not been established [26,27]. MPTP is a neurotoxin that selectively destroys dopamine neurons [28]. Currently, MPTP is widely applied in the establishment of PD animal models; while *in vitro* experiments, SH-SY5Y cells were cultivated in 1 mM MPP^+^ for 24 h to establish PD model. However, the concrete molecular mechanisms of MPP^+^ induced neuronal damage need to be further elucidated.

LncRNAs were evidenced to be dysregulated in many diseases, and they may be potential targets for disease diagnosis and treatment [29]. Recently, lncRNAs have been reported to be interrelated to the progression of PD via delivering molecular mechanisms. For instance, lncRNA H19 attenuates apoptosis in MPTP-treated PD through mediating miR-585-3p/PIK3R3 [30]. Reports from Zhao et al. revealed that lncRNA HOTAIR accelerated MPP^+^ stimulated neuronal damage in PD through miR-874-5p/ATG10 axis [11]. Studies have demonstrated that lncRNA BACE1-AS is up-regulated in PD [12], but its specific role and molecular mechanism remains unclear. The lncRNA/miRNA/mRNA axis has been reported to be associated with lncRNA pathogenesis primarily in many diseases, including PD. MiR-214-3p is a significant oncogene in various prevalent cancers. Over-expression of miR-214-3p is related to clinical development and poor prognosis of diseases [31]. Previous reports have revealed that lncRNA BACE1-AS sponges to miR-214-3p play roles in AD by regulating miR-214-3p [32]. Moreover, miR-214-3p has been reported to be down-regulated in PD. Thus, we assumed that lncRNA BACE1-AS may be associated with PD through mediation of mir-214-3p expression. The flow sheet abstract of this study was shown in Supplementary Figure 1.

Firstly, SH-SY5Y cells were stimulated with 1 mM MPP^+^ for 24 h to establish PD model *in vitro* and then the lncRNA BACE1-AS and miR-214-3p levels in MPP^+^-stimulated SH-SY5Y cells were determined using qRT-PCR. We observed that lncRNA BACE1-AS was over-expressed in MPP^+^ stimulated SH-SY5Y cells, and miR-214-3p was under-expressed in SH-SY5Y cells, indicating that lncRNA BACE1-AS was involved in development of PD through targeting of miR-214-3p. Herein, we speculated that there may be a functional relationship between the abnormal expressions of lncRNA BACE1-AS and MPP^+^ stimulated SH-SY5Y cells functions in PD progression. To verify our hypothesis; control-siRNA, lncRNA BACE1-AS-siRNA, inhibitor control, and miR-214-3p inhibitor were added into SH-SY5Y cells for 48 h. We found that lncRNA BACE1-AS-siRNA remarkably inhibited lncRNA BACE1-AS expression and increased miR-214-3p level, while the functions of lncRNA BACE1-AS-siRNA were blocked by miR-214-3p inhibitor. Furthermore, miR-214-3p level was suppressed in miR-214-3p inhibitor group. In summary, our observations implicated that miR-214-3p inhibitor interfered with lncRNA BACE1-AS functions in PD.

Moreover, we proposed that lncRNA BACE1-AS regulated the MPP^+^ stimulated SH-SY5Y cell function through mediation of miR-214-3p. This observation was further verified by lncRNA BACE1-AS-siRNA partly abolishing the roles of MPP^+^ in SH-SY5Y cells; including cell viability, apoptosis, LDH release, oxidative stress, and inflammation, while these effects of lncRNA BACE1-AS-siRNA was further eliminated by miR-214-3p down-expression. Having investigated the relevance between lncRNA BACE1-AS and miR-214-3p, we analyzed the latent mechanisms of miR-214-3p in SH-SY5Y cells. Based on TargetScan and dual-luciferase reporter analysis, we provided evidence for direct interaction of CDIPI with miR-214-3p. CDIP1, an important p53/TP53-apoptotic effector, was reported to regulate TNF-α-mediated apoptosis in a p53/TP53-dependent manner [33]. Our findings further illustrate that lncRNA BACE1-AS has a protective effect on PD by regulating miR-214-3p/CDIP1 signaling axis. After MPP^+^ treatment, we found that MPP^+^ significantly enhanced CDIP1 expression in SH-SY5Y cells. Further qRT-PCR analysis illustrated that miR-214-3p negatively regulated CDIP1 levels in SH-SY5Y cells. We observed that CDIP1 was up-regulated in CDIP1-plasmid transfected SH-SY5Y cells, in comparison with mimic control. In addition, miR-214-3p mimic drastically enhanced the mRNA levels of miR-214-3p and reduced CDIP1 expressions in SH-SY5Y cells, compared to the mimic control group. However, we observed the opposite results in CDIP1-plasmid group, as evidenced by enhanced CDIP1 mRNA levels and protein expression. Neuronal apoptosis, inflammation and oxidative stress are three vital factors in tissue injury [34]. Further analyses in this study revealed that CDIP1-plasmid inverted the functions of miR-214-3p mimic in SH-SY5Y viability, apoptosis, oxidative stress, and inflammation.

However, there were also some limitations of current study. For example, this study did not explore the role of lncRNA BACE1-AS in the PD animal models. And whether the expression of lncRNA BACE1-AS/miR-214-3p has a certain correlation with the clinicopathological parameters of PD patients was also not analyzed. We will explore this in depth in our next study.

## Conclusion

Our findings demonstrate that lncRNA BACE1-AS regulates oxidative stress in inflammatory response and neuronal apoptosis by directly mediating miR-214-3p/CDIP1 signaling axis, and thus, is associated with the occurrence and progression of PD. Further, lncRNA BACE1-AS may be a powerful candidate for PD diagnosis, and the lncRNA BACE1-AS/miR-214-3p/CDIP1 axis may be a prospective therapeutic candidate for PD. Our research provides new ideas for the diagnosis and treatment of PD.

## Supplementary Material

Supplemental Material

## Data Availability

The datasets used and/or analyzed during the current study are available from the corresponding author upon reasonable request.
