# Supplementary figures and images for: Long noncoding RNA BACE1-antisense transcript plays a critical role in Parkinson’s disease via microRNA-214-3p/Cell death-inducing p53-target protein 1 axis
Source: Bioengineered. 2022 Apr 28;13(4):10889–901. doi: 10.1080/21655979.2022.2066750 (PMC9208522; doi:10.1080/21655979.2022.2066750)

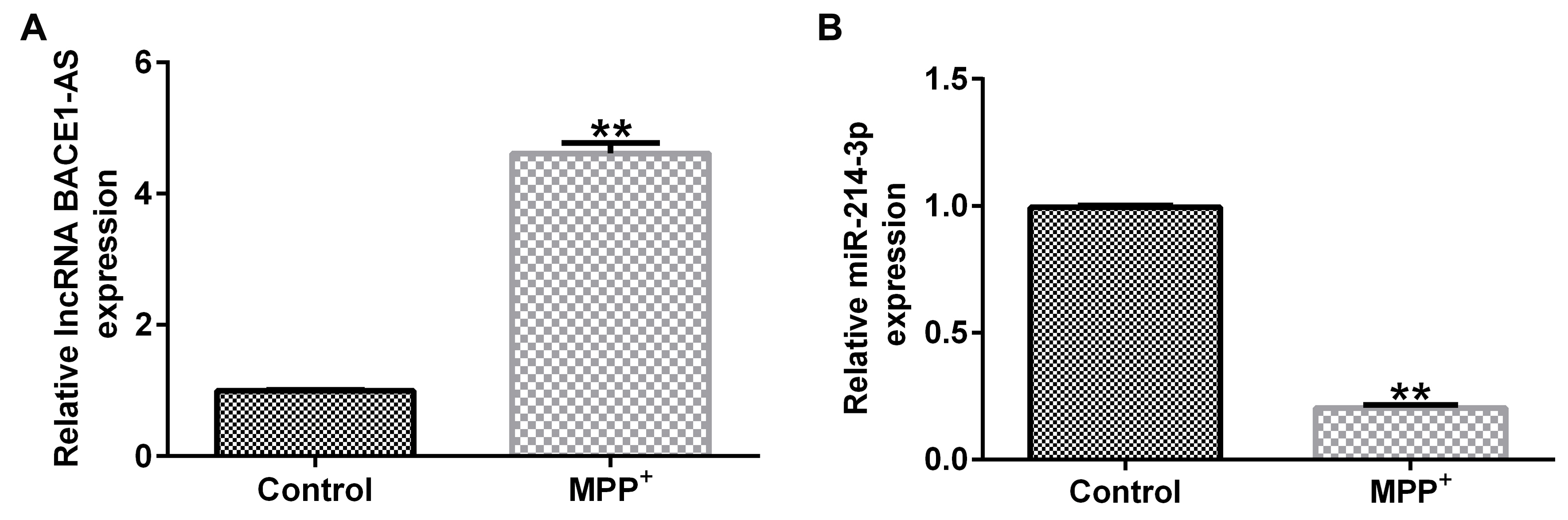

Supplement: Supplemental Material [file KBIE_A_2066750_SM8554.zip › supplementary/Supplementary Figure 1.tif]
