# Supplementary material for: Long noncoding RNA BACE1-antisense transcript plays a critical role in Parkinson’s disease via microRNA-214-3p/Cell death-inducing p53-target protein 1 axis
Source: Bioengineered. 2022 Apr 28;13(4):10889–901. doi: 10.1080/21655979.2022.2066750 (PMC9208522; doi:10.1080/21655979.2022.2066750)
Supplement: Supplemental Material [file KBIE_A_2066750_SM8554.zip › supplementary/Supplementary materials.zip!/Supplementary materials/original blots-Western blot assay/Notes.docx]

Dear editor,

Thank you so much for everything you have done for our manuscript. We have provided the original blots as supplementary document for reviewing. And it should be noted that during the Western blot assay, we first cut out the corresponding membrane according to the molecular weight of the target protein and then incubate the primary antibody. Therefore our original imprint is not a full film. We guarantee that all the experiments are carried out scientifically and strictly in accordance with the experimental procedures. Hope to get your understanding and support. We hope this is acceptable, otherwise we will redo this part of the experiment as requested.
